# Annual Commencement of Rush Medical College

**Published:** 1852-03

**Authors:** 


### ARTICLE VII.

### *Annual Commencement of Rush Medical College.*

The ceremony of confering the Degree of Doctor of Medicine by this Institution, took place in the City Hall on the 19th of Feb. ultimo, before a crowded assembly of citizens.

The valedictory address by the President of the Institution, Prof. Brainard, was upon Resuscitation as a legitimate aim and object of research. He treated the subject in that comprehensive style and masterly manner which generally mark his public efforts. We are sorry he has declined an application from the Class for a copy of the address for publication.

We append a list of the names of the young gentlemen who received the Degree with the title of their theses.

| *Names.* | RESIDENCES. | THESES. |
|---|---|---|
| Wm. C. Hunt, | Ills. | Human Ovology. |
| Vincent L. Hurlbut, | Ills. | On Scarlatina, [man Species. |
| Lewis D. Martin, | O. | Development and decay of the Hu- |
| Wm. D. Craig, | Ills. | Malignant Growths. |
| Orvis S. Johnson, | Ills. | On Intermittent Fever, |
| J. H. Reeder, | Ills. | On Dysentery. |
| Abram H. Knapp, | N. Y. | On Inflammation. |
| Franklin Blades, | Ills. | On Fever. |
| F. M. Crouse, | Ind. | On Arsenious Acid. |
| Isaiah P. Lynn, | Me. | On Contagion, |
| Benjamin T. Buckley, | Ills. | Phthisis Pulmonalis, |
| Geo. A. Bodenstab, | Ills. | Febres Intermittentes. |
| Jeremiah Youmans, | Wis. | On Menstruation. |
| Dudley Rodgers, | Ind. | On Etiology. |
| Stephen C. Gillett, | Ills. | Signs of Pregnancy. |

| | RESIDENCES. | THESES. |
|---|---|---|
| M. M. Hoozen, | Ills. | On Respiration. |
| A. B. Chadwick, | Mich. | On Quinine. |
| Hiram C. Jones, | Ills. | On Typhoid Fever. |
| Wm. M. Hobbie, | Ills. | Indications of treatment in Inflammation. |
| Alexander DeArmand, | Mich. | On Menstruation. |
| Edwin R. Williard, | Mich. | Philosophy of Nervous Powers. |
| G. J. Bently, | Ind. | On the Blood. |
| Walter R. Godfrey, | Ind. | On Chemistry. |
| Henry D. Adams, | Wis. | On Digestion. |
| Hugh Marshall, | Ills. | On Intermittent Fever. |
| John D. Woodworth, | Mich. | On Acute Rheumatism. |
| L. D. Tompkins, | Mich. | Report of cases in Practice. |
| T. G. Cole, | Ills. | Periodic and Continued Fevers. |
| A. F. St. Sure Lindsfelt, | Wis. | Therapeutiques De La Terebinthine. |
| Ezra M. Light, | Ills. | Hygiene and Prophylaxis. |
| Ezra Van Fossen, | Ind. | Acute Hepatitis. |
| M. G. Parker, | Ind. | Typhoid Fever. |
| Wm. H. Davis, | Ills. | On Diseases of Children. |
| H. A. Johnson, | Ills. | Malignant Heterolagous Tissues. |
| John Garrison, | Ills. | On Surgery. |
| Geo. W. Albin, | Ills. | On Uterine Hemorrhage. |
| James A. Collins, | Ind. | Epidemic Dysentery of Hendricks Co., Ind., in 1850. |

